# The Offering, Scheduling and Maintenance of Elective Advanced Pharmacy Practice Experiences

**DOI:** 10.3390/pharmacy3040355

**Published:** 2015-12-04

**Authors:** Rex O. Brown, Zalak V. Patel, Stephan L. Foster

**Affiliations:** Department of Clinical Pharmacy, College of Pharmacy, University of Tennessee Health Science Center, Memphis, TN 38163, USA; E-Mails: zalvpate@uthsc.edu (Z.P.); sfoster@uthsc.edu (S.F.)

**Keywords:** pharmacists, experiential education, electives, advanced pharmacy practice experience

## Abstract

The Accreditation Council for Pharmacy Education (ACPE) provides standards for colleges of pharmacy to assist in the provision of pharmacy education to student pharmacists. An integral part of all college educational programs includes the provision of experiential learning. Experiential learning allows students to gain real-world experience in direct patient care during completion of the curriculum. All college of pharmacy programs provide several Advanced Pharmacy Practice Experiences (APPEs), which include a balance between the four required experiences and a number of other required or elective APPEs. Required APPEs include advanced community, advanced institutional, ambulatory care, and general medicine. The elective APPEs include a myriad of opportunities to help provide a balanced education in experiential learning for student pharmacists. These unique opportunities help to expose student pharmacists to different career tracks that they may not have been able to experience otherwise. Not all colleges offer enough elective APPEs to enable the student pharmacist to obtain experiences in a defined area. Such an approach is required to produce skilled pharmacy graduates that are capable to enter practice in various settings. Elective APPEs are scheduled logically and are based upon student career interest and site availability. This article describes the offering, scheduling and maintenance of different elective APPEs offered by The University of Tennessee College of Pharmacy.

## 1. Introduction

Pharmacy schools offering professional programs in the US leading to the Doctor of Pharmacy (PharmD) Degree are required to meet several standards to achieve and maintain accreditation. Those standards are developed by the Accreditation Council for Pharmacy Education (ACPE). Every few years, ACPE provides updates to improve its standards to allow for better ways to evaluate the consistency, efficiency, and effectiveness of the accreditation activities and evaluations [1]. These standards are developed to assure and advance quality in pharmacy education, as well as to reflect the expectations that the US Department of Education has on the colleges and schools offering a professional pharmacy degree [1]. One of the ACPE standards includes advanced pharmacy practice experiences (APPEs) in the curriculum. According to ACPE Standard 13, there must be required and elective APPEs that advance and assess knowledge, skills, and professional attitudes and behaviors [1]. This paper describes the offering, scheduling, and maintenance of elective APPE offerings at a public college of pharmacy.

## 2. College of Pharmacy Curriculum

At the University of Tennessee College of Pharmacy (UTCOP), completion of 11 calendar-month APPEs is required for graduation. The 1760 h of APPE experience exceeds the minimum of 1440 h in ACPE Standard 13 [1]. In addition to the four required APPEs, seven APPEs are electives. The student may choose from a variety of options based on their personal and professional interests and availability from the APPE sites [2,3]. Our curriculum requires each student to complete eleven 1-month APPEs which include a minimum of 160 h per APPE. The last one and half professional years of the curriculum are comprised of completion of two two-week introductory pharmacy practice experience (IPPE) rotations, 11 APPEs, one month of didactic electives, one monthly comprehensive pharmacy update elective, and one month off. The experiential learning requires active participation of the student pharmacist as opposed to passive learning that occurs in many didactic courses. The student pharmacists gain practical, real-world experiences through their APPEs. Thinking and problem-solving skills, pharmacotherapy knowledge, and professional maturation are positive byproducts of the APPE experience. The UTCOP incorporates acute care, chronic care, and wellness through promotion of patient care services in community, ambulatory and healthcare-system settings. During APPEs, students gain in-depth experiences that allow them to be exposed to a variety of pharmacy practices and to experience different fields in pharmacy [2]. The UTCOP offers 43 different elective APPEs for the student pharmacists to choose from.

## 3. Introductory Pharmacy Practice Experiences (IPPEs)

Students are engaged in planning their APPE schedule during the fall semester of the 3rd professional year. Before the students begin their APPEs, completion of the IPPE program which is 335 h must be completed. The IPPE program includes two two-week rotations. One of the two-week experiences is in a community pharmacy, and the other two-week experience is in a healthcare system. IPPE exposes students to common contemporary practice models, professional ethics and expected behaviors, and direct patient care activities [4]. They are designed to help develop an understanding of what is expected for future APPEs. For this reason, students may choose to complete their IPPE rotations during the summer following the 1st or 2nd professional year. They may also opt to start the IPPE in the spring of the P3 year immediately before starting the APPEs.

## 4. Advanced Pharmacy Practice Experiences (APPEs)

As the 3rd year student pharmacists assist in APPE choices for their schedules, they are given the option to emphasize different career tracks. If a student pharmacist has a field of pharmacy that they would like to pursue, this is where they can choose an appropriate career track for their APPEs. The two tracks that the students can choose from are community pharmacy or inpatient residency health-system pharmacy. The community track is designed to lead towards community pharmacy employment or a community pharmacy residency. The inpatient track is designed to lead one towards an acute-care residency in health-system pharmacy. The resultant assignment to a residency requires a successful match in the American Society of Health-System Pharmacists Program in the spring of the P4 year. These APPE career tracks are optional (Table 1). Many students are unsure of their career direction and opt for a well-rounded schedule of APPEs, so a decision on career direction can be made later while gaining knowledge and experience as they complete the APPEs. However, the selections of these elective APPEs can define how student pharmacists look on “paper” as they compete for employment in the workplace or post-graduate education [5].

**Table 1:** Advanced Pharmacy Practice Experiences (APPEs) for the two career tracks.

Community Pharmacy Track (Example)	Inpatient Residency Track (Example)
Advanced Community Pharmacy (chain)	Second Medicine
Advanced Community Pharmacy (independent)	Critical Care
Community Pharmacy Management	Infectious Disease
Medication Therapy Management	Cardiology
Non-sterile Compounding	Nutrition Support
	Pediatrics
	Long-term Care

Listed are a few examples of elective APPEs that can be taken to follow the respective career track.

### 4.1. Elective APPEs

The required APPEs serve to keep a balanced schedule by exposing all student pharmacists to four key areas: ambulatory care, health-system pharmacy, community pharmacy, and general medicine. In UTCOP’s curriculum, this leaves seven APPEs in the elective category. The major categories of the elective APPEs are: health-system, community, state organizations, federal government, and International. In order to keep a balance among all the areas and give a fair chance of assignment to all students, there are a certain number of APPEs that a student may sign up for in each category. Also, all experiential learning programs face barriers that will likely limit the number of APPEs in each category [6,7]. For instance, all students must complete at least seven APPEs in direct patient care. Three of these are in the required APPEs (general medicine, advanced community, ambulatory care). This ensures that student pharmacists gain a variety of experiences and become a well-rounded pharmacist.

#### 4.1.1. Health-System Elective APPEs

Traditional health-system elective APPEs include several direct patient care experiences in a hospital/healthcare setting. Students are given the opportunity to expand their abilities in pharmacotherapy management and inter-professional team-based care serving diverse patient populations. Examples of the traditional health-system elective APPEs include infectious disease, nephrology, mental health, emergency medicine, cardiology, pulmonology, nutrition support, trauma and critical care. Institutional APPEs often focus on a specialized population which may include oncology, gerontology, neonatology and palliative care patients. Traditional institutional elective APPEs are taught by both part-time and full-time faculty members.

Traditional APPE elective sites provide opportunities for student pharmacists to attend daily patient rounds with a medical and/or pharmacy team. While on rounds, student pharmacists are required to collect patient data, monitor lab results, develop drug therapy plans, and communicate with patients and caregivers about different treatment options [8]. They may also be required to refer cases to clinical pharmacy specialists, answer medication-related questions and perform patient counseling. Student pharmacists get a chance to review and learn hospital protocols and formularies to assist them in developing a therapy plan to treat a patient. These are critical skills for a pharmacist to have while working in any pharmacy environment.

In addition to traditional institutional APPEs, there are non-traditional APPEs offered as electives that focus on other pharmacy services. These APPEs tend to not focus on direct patient care and include experiences in institutional management, pharmacy informatics and medications safety The UTCOP has labeled these APPEs as other professional experiences (OPEs). The nuclear pharmacy APPEs are also included in this category and are generally offered to those student pharmacists who have completed the certificate program in this area. A list of the institutional elective APPEs is provided in Table 2. These elective APPEs allow students to engage in the operational services in a healthcare system setting and to understand the rules and regulations of accrediting organizations. Institutional management APPEs are usually offered by directors of pharmacy. The focus of this APPE includes responsibilities of the director such as how the department of pharmacy integrates with the institution or healthcare system. The Informatics APPE elective requires learning about the application of technology and information systems by pharmacists in the medication-use process for the purpose of improving health outcomes. It focuses on the importance of the knowledge of both pharmacy practice and informatics. Medical information and medication safety would help evaluate the medication delivery system used in the healthcare setting for medication errors and prevention strategies.

**Table 2:** Traditional and Non-traditional institutional elective APPEs.

Direct Patient Care Traditional	Other Professional Experiences within the Healthcare System	Other Professional Experiences outside the Healthcare System
Emergency Department Transplant Nephrology Palliative Care Gerontology Neonatology		
Oncology Chemical Dependence	Medical Information Clinical Institutional Management	Pharmacogenomics Clinical Research Clinical Toxicology Association Management
Critical Care Infectious Disease Long term care Cardiology Nutrition Support Pediatrics	Specialty Pharmacy Institutional Management Informatics	Managed Care in Prison Pharmacy Veterinary Pharmacy
Home Infusion Pediatric Asthma HIV	Medication Safety Nuclear Pharmacy	Academia

This is a detailed list of elective APPEs offered at UT College of Pharmacy. They are primarily in an ambulatory or a hospital/inpatient setting.

#### 4.1.2. Community Electives

The second category of the elective APPE includes those in the community setting. There are a variety of independent, chain and food-store pharmacies that offer good learning opportunities for student pharmacists. Some of the elective APPEs in a community setting include the following: medication therapy management (MTM), compounding, promotion of wellness, community management, and a second advanced community. Elective APPE experiences at these sites permit students to see the commercial-management side of the pharmacy which is different from the traditional community pharmacy experience. In traditional community pharmacy, students actively engage in duties pertaining to daily functioning of the pharmacy. This may include, but not limited to, conducting patient interviews/medication histories, processing new prescriptions, compounding, dispensing medications, giving immunizations and counseling patients on devices and nonprescription medications.

In addition to the role of the pharmacist in a community setting, students are able to see how pharmacy technicians, insurance companies and manufacturing companies play a role in the community healthcare system. This setting often exposes student pharmacists to a “collaborative” model which includes daily interaction with patients and health-care practitioners [9]. Practicing to research and retrieve medical information through different sources and effectively communicating face-to-face is an important skill in the community setting. In addition to patient interaction, students are given the opportunity to effectively and professionally communicate with pharmacy personnel and other healthcare professionals [10]. At times, they will have to retrieve an appropriate resource for medical information when providing information to patients or other healthcare professionals.

Medication therapy management is another focus for the student pharmacists in the community setting. Students can play a major role in managing medications and collaborating with physicians and other healthcare professionals to optimize medication use. The MTM service model in pharmacy practice includes the following five core elements: medication therapy review (MTR), personal medication record (PMR), medication-related action plan (MAP), intervention and/or referral, and documentation and follow-up [11]. Students screen patient profiles and check for items such as adherence to guidelines, immunizations, drug-disease contraindications, duplicate therapy, and high-cost medications that can be converted to a generic. In addition, students learn how to perform a Comprehensive Medication Review (CMR). In the CMR, student pharmacists demonstrate to patients the proper techniques using their medication devices such as insulin pens, testing supplies, and inhalers. They provide information on what each medication is used for and what to monitor for in terms of adverse drug reactions. An important component of MTM is to understand the patient population’s educational level so communication can be delivered comprehensively and effectively. Community APPE electives would be a great chance for the student pharmacist to learn more about the future of being a provider and to see the possible changes that the future will bring.

Corporate pharmacy is another area that can emanate from the community setting APPE offerings. This APPE elective allows students to gain knowledge in the administrative side of community pharmacy. For students who desire to engage in a career path to advance within the corporation, this elective could be an excellent opportunity. This could result in building relationships with local pharmacists and district/regional pharmacy leaders, resulting in leadership opportunities in the future.

#### 4.1.3. State Organization Electives

The third category of the elective APPEs includes experience at state and private organizations. These experiences offer a completely different perspective of the pharmacist’s role. Organizations such as TennCare (State of Tennessee Medicaid Program alternative), Cigna, Blue Cross Blue Shield of Tennessee, Tennessee Pharmacists Association (TPA), and the State Board of Pharmacy, conduct APPEs that allow student pharmacists to experience the pharmacists’ role in the state or federal government and the private sector. Students can see how different pharmacy laws and regulations are enforced. Working with the insurance companies, students are given the chance to see how insurance policies and formularies work and impact pharmacy practice.

#### 4.1.4. Government Electives

There are several elective APPE opportunities within the Army, Air Force, and Indian Health Services. The UTCOP has secured APPE electives in Alaska, New Mexico, Arizona, Wyoming, and Kentucky. All of the listed states, except Wyoming, provide a variety of experiences in different settings, such as ambulatory care, community, hospitals, and clinics. The elective in Wyoming focuses on ambulatory care and community practice. Students who do not mind living in rural areas may find such electives as a good fit for them to offer healthcare to an underserved population (e.g., Indian Health Services). Approximately 50 student pharmacists complete a government APPE each year.

#### 4.1.5. International Electives

UT College of Pharmacy offers 4th year student pharmacists the opportunity to complete a month-long elective APPE in another country. Student pharmacists from our college have completed international APPE electives in Australia, New Zealand, England, France, Japan, Hungary, Ireland, Scotland, Spain, Sweden, Thailand, and Turkey. The student pharmacists are able to learn about pharmacy practice and healthcare systems in the respective countries. They can then compare them to those in the United States. In addition to healthcare systems, students can learn about the culture and social life of the respective country. Approximately 30 students complete an international elective APPE each year. This program has been in place for over 20 years and continues to grow at a controlled pace.

In order for students to take an international elective APPE, the college must have a formal affiliation agreement with the host institution. In return, the college allows students from that affiliated institution to come to the United States to experience pharmacy practice. These international APPEs have been identified as life-changing experiences by some student pharmacists who have completed them.

## 5. Benefits of Offering a Variety of Electives

We feel that our student pharmacists receive extensive benefits from participation in the elective APPEs. First, they provide real-world opportunities that are necessary for the student pharmacist to become knowledgeable, skilled, and caring pharmacists and to provide pharmaceutical care [12]. Given the variety of choices over 15 months, student pharmacists are able to steadily improve their skills. Eventually, the student pharmacists are able to become licensed through successfully passing the board exams, NAPLEX and Multistate Pharmacy Jurisprudence Exam (MPJE). It is unknown whether a variety of elective APPEs directly help student pharmacists pass the NAPLEX and MPJE. Second, as a result of the experiential learning activities that are designed to consistently challenge students to attain higher levels of performance, students are able to strengthen their professional confidence [10]. Ultimately, the graduates are prepared to enter the next phase of their career.

About 80% of our students who applied for post-doctoral training in 2015 matched for a residency, fellowship, or graduate program. The remainder of the graduates was able to secure permanent pharmacy employment. Since the introduction of the community APPE track three years ago, the percentage of students opting for this career path has increased from 4% to 9% to 19%. The exit survey of the last graduating class reported that 99.2% of the class strongly agreed or agreed that they were ready to enter pharmacy practice. In our latest preceptor survey (2014), 94% agreed or strongly agreed that our student pharmacists could develop and use patient-specific pharmacy care plans. Additionally, 98.2% of the preceptors agreed or strongly agreed that our student pharmacists maintained professional competence. Based on these data, we feel that the elective APPEs have an overwhelmingly positive effect on our student pharmacists as they pursue employment or residency training. Students who perceive satisfaction with their experiential learning are also more likely to have a positive view of their overall academic experience, and thus, serve as active alumni and as a future preceptor faculty member. This allows them to be involved in the clinical training and help mentor future pharmacists [6].

## 6. Quality Assurance for Maintenance of Experiential Learning

Student activities during these APPEs take place under the supervision and monitoring of a qualified preceptor. Our school has established a formal process for the appointment of these skilled preceptors. They are, in most cases, licensed pharmacists and meet the requirements to be a preceptor. A newsletter is published four times a year announcing any new preceptors appointed. To train the new and the current preceptors, our college hosts a preceptor development conference each year in three different cities. These programs are offered to around 120 preceptors in Memphis, Nashville, and Knoxville.

In addition, substantial recognition is given to the preceptor faculty. The recognition is given based on effective mentorship, professionalism, and care and empathy for patients, as well as, the student pharmacist training [5,9]. Each year we recognize three preceptors in our newsletter. This is called the Preceptor Spotlight and a preceptor from east, middle, and west Tennessee is featured each year. Four other preceptors are honored each year on Rotation Day. One preceptor of the year for IPPE and three preceptors of the year for APPE (one for each region of the state) are recognized. Regular site visits are conducted across the state to help ensure quality in the experiential learning program.

## 7. Conclusions

The purpose of APPEs is to provide learning experiences to help students explore career areas and prepare them for future employment and independent living. They gain basic skills, thought processes, and professional qualities required for various areas of pharmacy practice. At the end, students are well-trained to compete and work at pharmacies in many different settings. We conclude that the elective APPEs contribute to the ultimate success of our graduates. Often the elective APPEs create a career direction for our graduates to pursue.
